# Mobile Real-Time Grasshopper Detection and Data Aggregation Framework

**DOI:** 10.1038/s41598-020-57674-8

**Published:** 2020-01-24

**Authors:** Piotr Chudzik, Arthur Mitchell, Mohammad Alkaseem, Yingie Wu, Shibo Fang, Taghread Hudaib, Simon Pearson, Bashir Al-Diri

**Affiliations:** 1 The University of Lincoln, School of Computer Science, Lincoln, LN6 7TS UK; 2 The University of Lincoln, Lincoln Institute for Agri-Food Technology, Lincoln, LN6 7TS UK; 3 The University of Lincoln, School of Pharmacy, Lincoln, LN6 7TS UK; 4 State Key Laboratory of Severe Weather, Chinese Academy of Meteorological Sciences, Beijing, 100081 China

**Keywords:** Environmental impact, Computational science

## Abstract

Insects of the family *Orthoptera: Acrididae* including grasshoppers and locust devastate crops and eco-systems around the globe. The effective control of these insects requires large numbers of trained extension agents who try to spot concentrations of the insects on the ground so that they can be destroyed before they take flight. This is a challenging and difficult task. No automatic detection system is yet available to increase scouting productivity, data scale and fidelity. Here we demonstrate MAESTRO, a novel grasshopper detection framework that deploys deep learning within RBG images to detect insects. MAESTRO uses a state-of-the-art two-stage training deep learning approach. The framework can be deployed not only on desktop computers but also on edge devices without internet connection such as smartphones. MAESTRO can gather data using cloud storge for further research and in-depth analysis. In addition, we provide a challenging new open dataset (GHCID) of highly variable grasshopper populations imaged in Inner Mongolia. The detection performance of the stationary method and the mobile App are 78 and 49 percent respectively; the stationary method requires around 1000 ms to analyze a single image, whereas the mobile app uses only around 400 ms per image. The algorithms are purely data-driven and can be used for other detection tasks in agriculture (e.g. plant disease detection) and beyond. This system can play a crucial role in the collection and analysis of data to enable more effective control of this critical global pest.

## Introduction

**Figure 1:**
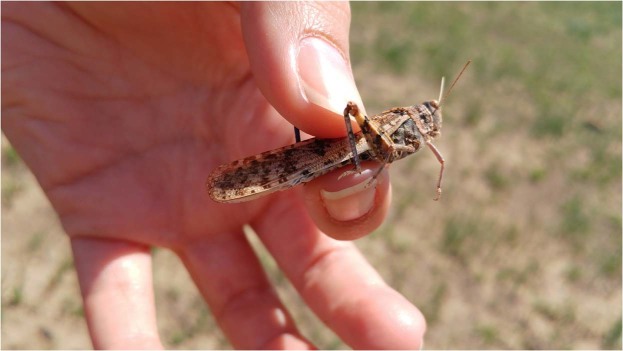
An adult grasshopper *Oedaleus asiaticus*.

Grasshoppers and related insects such as locusts (family *Orthoptera: Acrididae*) are pest insects and can damage crops and eco-systems (see Figure 1). While grasshoppers are usually seen as individuals, they can gather in large groups^1^ and devastate vegetation. Grasshopper swarming behaviour is uniquely found in both the nymph and adult stages^2^, when they reach the adult stage, they can swarm in the air^3^. They are highly diverse, in Inner Mongolia alone there are approximately 130 grasshopper species^4^, these vary within regional environmental sub zones. Of the 130 species there are three common types: the early-season species *Dasyhippus barbipes*, the mid-season species *Oedaleus asiaticus*, and the late-season species *Chorthippus fallax*^5,6^. This high diversity makes recognition of individual species challenging. In addition grasshoppers show phenotypic plasticity or polyphenism (multiple phenotypes arise from a single genotype), which is a density-dependent physiological phase that depends on environmental conditions^7^. Grasshoppers are also known to change their appearance and colour in response to changes in their social status and environmental stimuli and to adapt to environmental changes^7^. Diversity and their response to the surrounding environment makes it challenging to discriminate insects within typical environmental backgrounds (grasslands) and poses a significant challenge for the adoption of image analysis systems for pest detection.

Edelaar *et al*.^8^ investigated the colour change phenomenon in Azure Sand Grasshoppers and their ability to match the colour of the surrounding environment as a response to the risk of predation. They have reared 2 populations of grasshopper third stage nymphs in dark and light coloured rearing boxes, painted with black or white from inside and containing a layer of stones of the same colour. Images were taken for the last stage nymphs using a Pentax K-r camera with fixed flash settings and constant ambient lighting. The results showed that the grasshoppers adjusted their colours to match the surrounding environment, becoming paler or darker compared to a control population. However, it was notable that colour changed grasshoppers, were never as pale or as dark as the backgrounds in the boxes where they were reared; to conclude that, the plasticity response in grasshoppers was never sufficient to prevent detection with the RGB images.

For effective control, the insects need to be detected on the ground before they start to develop air borne swarms. Detection systems need to determine pest density and location with high speed and accuracy. Location of the swarms on the ground then enables their control by the application of pesticides^9^ and bio-pesticides. However, there are some drawbacks to these measures, such as the need to find the pest at a young age for maximum effectiveness and potential adverse environmental and social impacts from the pesticides. Currently, the detection of grasshoppers is a manual operation conducted by extension agents or farmers. This is an expensive and frustrating task since the pest can be located across vast areas of remote land and requires highly trained staff who need to enumerate large numbers of mobile grasshoppers occluded by vegetation.

The development of locust image recognition systems is in its infancy but previously Xiong *et al*.^10^ proposed a grasshopper segmentation algorithm using pulse-coupled neural network (PCNN); although the algorithm was tested using only two images which makes the fair comparison and evaluation impossible. In addition, this algorithm does not provide any bounding boxes annotations, hence it cannot be treated as a detection system. As noted by the paper’s authors: “the PCNN algorithm should be viewed as a preprocessor” that needs to be combined with other image processing transforms to be a complete system. Other notable attempts to using computer vision to detect and classify pests include Ziyi *et al*.^11^ employing a saliency map and deep convolutional neural network (DCNN) learning for localization and classification of insects on internet images. While achieving good results, their data set was mined from the internet and all example images given were of high-quality images with a large contrast of colour between the insect and background. This is a very different set of circumstances to the detection of insects in the natural environment where the background image layer can be highly diverse. Images of insects in the natural environment usually includes diverse image qualities and well-camouflaged/occluded grasshoppers. In addition their^11^ method was conducted on a computer with a powerful graphics card and CPU that may not be easily available to a wide diversity of human operators. Liu *et al*.^12^ used a novel detection network to detect a number of pests in laboratory settings. Their algorithm achieves 75% mean accuracy precision for their bounding boxes. While having impressive results the photos were taken in laboratory conditions once again have low background noise, making it easier to detect the insects. The only other insect detection using region-based convolutional neural networks (RCNN) was^13^ who use Fast RCNN to detect various insects in images. They achieved 89% accuracy on their detection challenge using a fixed image size of 450 × 750 pixels from a non referenced data-set mined from the internet rather than collected from the wild. The proposed work uses one stage for both region proposal and classification, which is more efficient than two-stage approach used in Fast RCNN. An experiment with a similar premise to Xia^13^ is Ding *et al*.^14^ approach to detect moths. They deployed a neural network detection algorithm using a sliding window over the entire image to identify potential objects. The proposed approach uses anchor boxes to process the whole image in a single iteration. Moreover, Ding *et al*. used images with artificial white background and high contrast with minimal interference that made the detection process easier.

This work proposes a mobile real-time grasshopper detection framework called MAESTRO (Mobile reAl-timE graSshopper deTection fRamewOrk). Compared with existing remote sensing systems (e.g. satellite and aerial) that rely on expensive hardware and require wireless network communication, the proposed system is able to detect grasshoppers locally using a mobile device. The detection step is performed by the deep learning model executed on a smartphone without an internet connection. Furthermore, the mobile application provides data aggregation functionality and collects other crucial data such as temperature, soil moisture, wind speed, or solar radiation. As a result, as soon the internet data becomes available, the collected data will be gathered on a cloud database. This data can then forecast the grasshoppers movements and respond efficiently to prevent, or contain, a grasshopper outbreak. To the best of the authors’ knowledge this is the first fully automated mobile grasshopper detection framework. Moreover, we present this powerful stationary framework that can be used when computational resources are not limited. The main contributions of this study are as follows. First, we propose a mobile real-time grasshopper detection framework called MAESTRO that can be used on a standard smartphone. Second, we present a novel stationary grasshopper detection framework which is the first fully automated grasshopper detection framework which includes a novel two-stage training approach for detection tasks. Finally, we present a new dataset for detection of grasshoppers called the GHCID (GrassHopper deteCtIon Dataset) that will facilitate the development of new algorithms for further study and changes to this field.

## Results and Discussion

To validate our models we prepared two sets of experiments. During the first set of experiments we tested both models capabilities to detect grasshoppers. In the second set, we present the mobile real-time detection framework. The implementation was based on Keras deep learning framework^15^ and Tensorflow numerical computation library^16^. The experiments were conducted using a Huawei Mate 20 Pro smartphone and a PC with Intel Core i7-6700K CPU, NVIDIA TitanX and 2080 Ti graphics cards, and 64 GB of RAM.

### Grasshopper Detection

**Figure 2:**
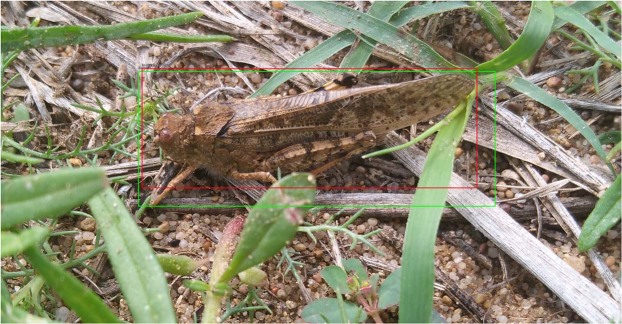
An adult grasshopper detected by the stationary framework. The green bounding box corresponds to the ground truth, whereas the red bounding box represents the detection generated by the stationary model.

To validate our approach we used the GHCID dataset. During experiments 4-fold cross-validation was performed, with each fold being treated as the testing set once. The 10% of the training set was treated as a validation set. As a result, we performed 4 experiments for each type of model with following split: 2414 images were used for training, 268 images were used for validation, and remaining 896 images constituted testing set. Early stopping criteria was used: training stopped when validation error did not improve for 10 epochs. Each training sample was subject to random artificial transformations (ATs) including rotation, shearing, scaling, photometric distortions, horizontal and vertical reflections with 0.5 probability. The ATs were performed to increase variety in the training set and combat overfitting; they were performed during models training so their computational footprint is limited. Finally, the *α* parameter of the weighted loss function was set to 0.75 meaning that the weighted loss function assigned 3 times more importance to the classification loss than the regression loss in the second stage of the training process. As mentioned before, the stationary model used a ResNet architecture as the backbone network. Compared with the original RetinaNet work^17^, that used ResNet with 101 layers, we decided to use a more compact ResNet architecture with 50 layers. The input images were re-scaled proportionally so that their dimensions are always between 800 and 1333 pixels. The model was trained using stochastic gradient descent (SGD) training algorithm with a batch size of 1 (due to input size and GPU memory constraints) and 0.0001 Adam optimization^18^. If learning error stops converging, the learning rate was reduced by 10% every 2 epochs. All training input-target pairs were shuffled between each epoch. To improve results, the values of the initial weights were transferred from a model trained on the ImageNet dataset^19^. All remaining details can be found in^17^. Figure 2 presents the example results generated by the stationary model.

The mobile model used Single Shot Detector (SSD) architecture with a weighted smooth L1 loss as the localization loss and a weighted sigmoid loss as the classification loss^20^. The input images were uniformly re-scaled to 300 × 300 pixels and grouped in batches of 12 for training. The MobileNetV2 architecture was used for feature extraction with 1.0 depth multiplier and RELU_6 activation functions used^21^. The network was trained using SGD algorithm with a batch size of 12 and RMSprop optimizer^20^. If learning error stopped converging, the learning rate was reduced by 10% every 2 epochs. All training input-target pairs were shuffled between each epoch. To improve results, the values of the initial weights were transferred from a model trained on COCO dataset^22^. All remaining details can be found in^20^.

**Figure 3:**
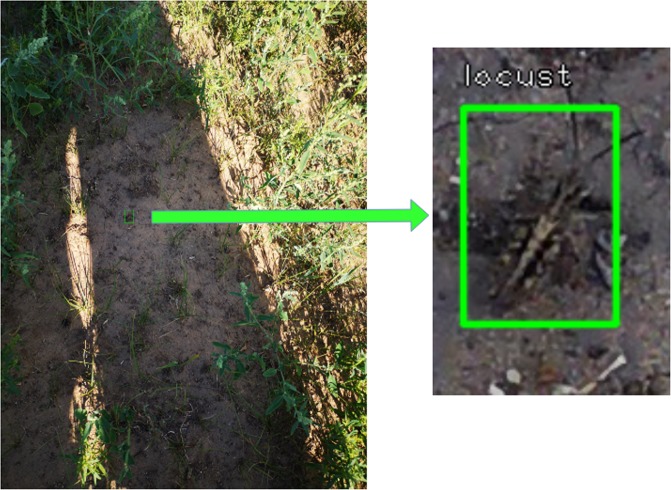
A grasshopper that was undetected due to poor lighting conditions (shadows) and far distance from the camera.

Table 1 presents the performance of both methods at different intersection over union (IoU) thresholds. The stationary method produces high mean average precision (mAP) for all major IoU thresholds. The main reasons behind the majority of errors are poor lighting conditions, obstacles obstructing grasshopper body and images being captured too far from grasshoppers. Figure 3 shows a grasshopper instance that is undetectable for a human expert.

**Table 1:** Performance comparison using mAP metric.

Method name	*IoU*_0.1_	*IoU*_0.25_	*IoU*_0.50_
Stationary Model (RetinaNet + ResNet-50)	0.758 ± 0.012	0.755 ± 0.011	0.701 ± 0.014
Mobile Model (SSD+MobileNetV2)	0.478 ± 0.013	0.454 ± 0.011	0.402 ± 0.011

As expected, the stationary method significantly outperforms the mobile method. The performance difference between the stationary and mobile models is caused mainly by the backbone network choice. The mobile framework uses a lightweight MobileNetV2 whereas the stationary model uses a Resnet-50 backbone which is more accurate thanks to more learning parameters. As such, Resnet-50 is not feasible to be used on a mobile device. The stationary method requires around 1000 ms to analyze a single image, whereas the mobile approach uses only around 400 ms per image using a PC. When evaluated using a mobile device, the quantized mobile approach requires 80 ms on average per image which results in 12.5 frames per second (FPS) that allows real-time detection. Unfortunately, the stationary model is too large to operate in any smartphone device. Table 2 presents the maximum recall values achieved by the proposed methods. Since in our particular case, we only care about finding all possible grasshoppers and we are not concerned with the misalignment of bounding boxes, the recall values were calculated for 0.01 IoU. As such, the presented values can be treated as the percentage of all grasshoppers that were successfully detected.

**Table 2:** Maximum recall values at 0.01 IoU.

Method name	*Recall*_*IoU* = 0.1_
Stationary Model (RetinaNet + ResNet-50)	0.783 ± 0.016
Mobile Model (SSD + MobileNetV2)	0.494 ± 0.014

### Mobile Application

**Figure 4:**
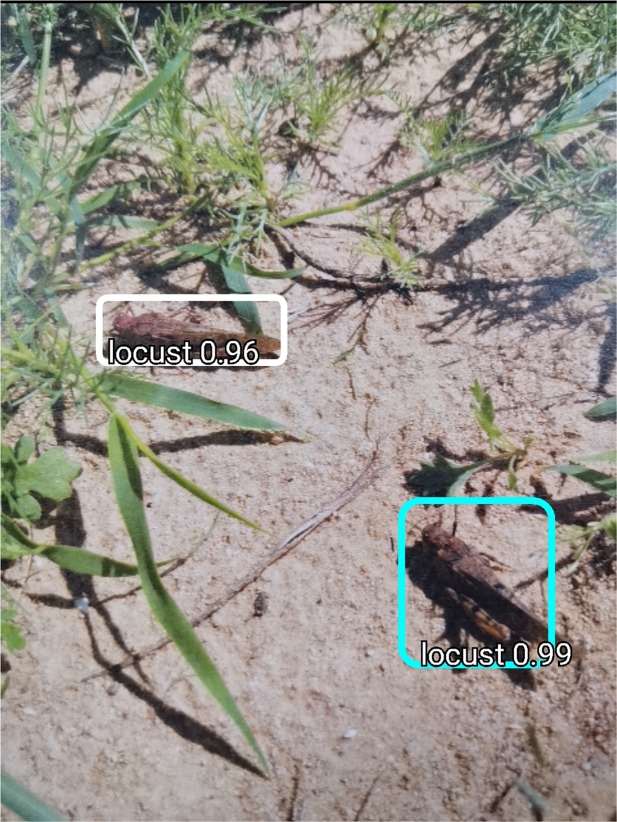
Grasshoppers found by the MAESTRO system. The numbers represent the framework’s confidence in detected objects being grasshoppers.

Figure 4 presents a working MAESTRO application. It was developed for Android devices, which are the most commonly used mobile devices around the world^23^. We used Tensorflow Lite (TL) framework which provides a set of libraries to execute deep learning (DL) models on mobile and embedded systems. The TL includes a number of hardware acceleration for DL models (e.g. Android Neural Network API) and automatically optimizes the model’s implementation for a specific device. Since the training procedure requires substantial computational resources that are unavailable on mobile devices, the model is trained on a PC and only the inference is performed using a mobile device.

Inference efficiency is the biggest challenge when deploying DL models to mobile devices. We decided to quantize our models to improve their latency and reduce the model’s size and computational footprint. The post-training quantization reduces the precision requirements for weights and activations from 32-bit to 8-bit which considerably speeds up the execution. This optimization provides very low latencies at the cost of tolerable accuracy degradation. The user can save any image together with other information locally on a device. Subsequently, the data can be sent to globally available GIS servers for further analysis of grasshopper movement and development. Most importantly, since location information is encoded directly into images, the user can use their own preferred map software to display and analyze the data. The collected data will be used for model re-training and continuous improvement of detection performance. The improved model will be send to all users as an application update.

## Conclusion

This paper presents a novel mobile real-time grasshopper detection framework called MAESTRO. The framework can be used on a standard mobile phone without an internet connection to detect and localize grasshoppers. Furthermore, it collects various crucial information about grasshoppers to facilitate an in-depth analysis of grasshoppers’ movement and development. The framework could be extended to consider other swarming insects such as the desert locust. Moreover, this work describes a stationary grasshopper detection framework that can be used for more accurate results when computational resources are not limited. This framework proposes a novel two-stage training approach for detection tasks that prioritizes the classification performance over bounding boxes accuracy. To the best of authors’ knowledge, these are the first fully automated grasshopper detection systems. Finally, we present a new and challenging dataset for grasshopper detection called GHCID to promote the development of new grasshopper detection algorithms by the community.

It is important to note, that although we designed the proposed methodology for grasshopper detection, the frameworks are purely data-driven and therefore can be used for other detection tasks in agriculture (e.g. plant disease detection) and beyond. In the future we plan to develop the system used for analyzing the collected data and include other data types such as satellite images. Additionally, we will combine the Feature Pyramid Network with the SSD architecture to increase the detection of grasshoppers at various scales. Finally, we are going to incorporate Online Hard Examples Mining technique^24^ to reduce the number of false positives generated by detection frameworks. Due to the modular design of both mobile and stationary frameworks it is relatively straightforward to change the backbone network responsible for feature extraction and improve detection performance. We strongly believe that the rapid development of mobile devices will facilitate the usage of more robust backbones and lead to improved performance.

## Methods

During this study, we created two separate grasshopper detection models each designed for different purposes: stationary and mobile. To produce these detection frameworks we decided to use deep learning algorithms, which are state-of-the-art machine learning techniques used for various computer vision and image processing tasks, including; detection^25^, segmentation^26^, classification, and localization of objects in pictures and videos^27^. The stationary model was designed to validate our methodological approach and provide baseline performance for the mobile model. It was created to achieve maximum performance when the computational environment is not restricted. The mobile model represents a compromise between speed and accuracy considerations. It was designed for mobile execution, optimized to perform continuous real-time detection and minimize application’s latency.

### Stationary Model

**Figure 5:**
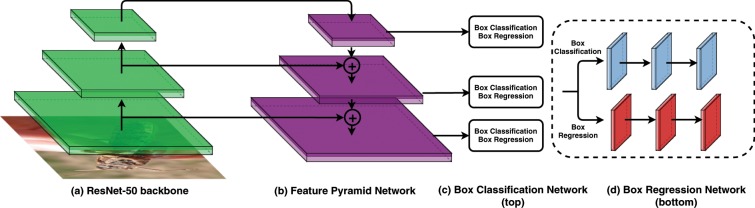
The RetinaNet architecture: The ResNet backbone network (**a**) together with the FPN (**b**) serve as the feature extractor, whereas two other CNNs are tasked with classification (**c**) and regression (**d**).

The dominant paradigm in the majority of object detection methods is a two-stage approach. The first stage generates all possible objects present in a scene, whereas the latter classifies them. Unfortunately, due to multiple levels of transformations that cannot be parallelized, this approach is slow to train and execute. Consequently, we decided to base our model on the RetinaNet architecture which is an example of a so-called *Single-Shot* detector^17^. As shown in Figure 5, the RetinaNet performs both tasks (region proposal and classification) at the same time, thus drastically reducing the computations needed.

**Figure 6:**
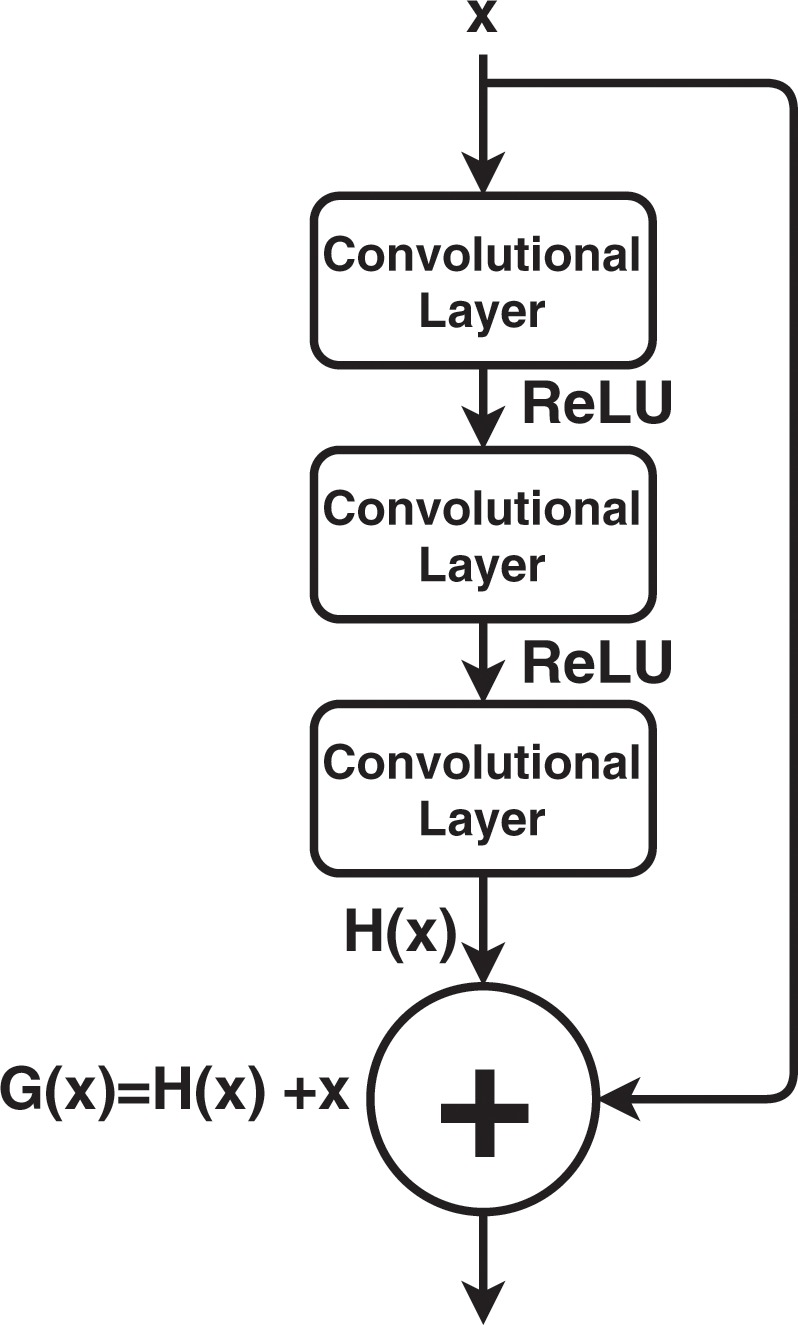
The residual block showing the identity shortcut connection (x identity) bypassing 2 transformation layers.

RetinaNet is composed of 4 dedicated deep convolutional neural networks (CNNs). The *backbone* network is responsible for extracting convolutional features from the input image. The final two CNNs are tasked with classifying the backbone network output and regressing it to a bounding box. Most importantly, they perform these tasks simultaneously. Furthermore, RetinaNet combines the Feature Pyramid Network (FPN) with the backbone network^28^. The FPN enhances the network’s architecture with lateral connections and the top-down pathway to construct multi-scale feature pyramids from a single input image. As a result, the RetinaNet is able to detect objects of different scales even though they might not have been seen during training procedure. Finally, RetinaNet introduces dedicated loss function called *Focal Loss* which is a variant of a cross entropy function. The Focal Loss forces the CNN to focus on training instances that it finds most difficult to classify. We decided to use the ResNet architecture as the backbone network due to it’s powerful representational ability and robust performance^29^. The core idea behind ResNet success is the introduction of so-called *identity shortcut connection* that bypasses one or more layers. A key advantage of this type of connection is that they allow direct training signal propagation between otherwise distant layers. As a result, they counteract the deterioration of a training signal and allow to build much deeper architectures. Figure 6 presents a residual block which is the fundamental building element of the ResNet architecture.

### Mobile Model

**Figure 7:**
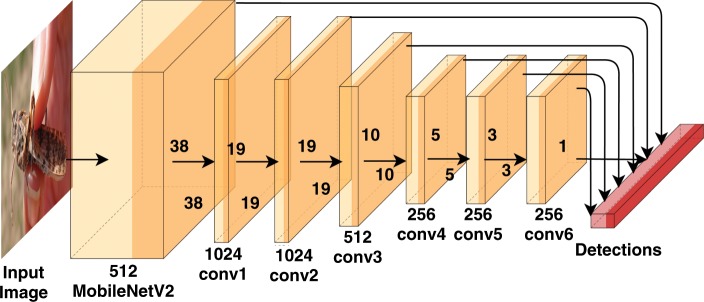
The SSD architecture.

The mobile model is based on the Multibox SSD. Similarly to the RetinaNet, the SSD network uses a base CNN to extract convolutional features. However, the bounding box regression and input classification are performed by another relatively small CNN (RetinaNet uses two dedicated CNNs). Figure 7 presents the SSD network, which uses multi-scale feature maps produced by intermediate layers of the base model to detect objects at various scales. This architecture is one of the fastest DL solutions for object detection that makes the real-time operation possible.

The MobileNetV2 was used as the base feature extractor^30^. This network architecture was specifically designed to work with computationally constrained environments. Compared with standard CNN, the MobileNetV2 uses depthwise separable convolutions which are much more efficient than standard convolutions but provide similar results. The MobileNetV2 introduced residual connections that allow training signals (gradients) to flow directly between indirectly connected layers.

### Training Procedure

In standard detection tasks, the accuracy of bounding boxes is as important as the accuracy of the classification process. In grasshopper detection (especially mobile), the classification performance has much more importance than bounding boxes’ accuracy. We focused more about finding grasshoppers, rather than ensuring the alignment of generated bounding boxes with ground truth boxes is correct. As a result, we propose a two-stage training procedure with a weighted loss function. In the first stage, we train the model until the early-stopping criteria is used. At this stage, the total loss function is calculated as the sum of the classification loss and the regression (bounding box related) loss. In the second stage, we fine-tuned the model using Stochastic Gradient Descent with Warm Restarts and cosine annealing learning rate (SGDR)^31^ and weighted loss function. A learning rate is the crucial hyper-parameter of the fine-tuning process. It determines the magnitude of weights updates during the training process. Since every problem domain is unique, there is no universal optimal learning rate. To train a robust model, the learning rate needs to be annealed to avoid fluctuations around the local minimum. However, the learning rate that is too small can almost stop the learning process. The SGDR proposes a new learning rate annealing procedure that is based on the cosine curve. It uses periodic changes to the learning rate, encouraging the model to transition from local minimums and navigate out of the troublesome parts of the weight space. We combine the SGDR with the weighted loss function (FL) that assigns more weight to the classification loss (CL) rather than regression loss (RL)

1 $$FL(\hat{y},y)=\alpha CL(\hat{y},y)+(1-\alpha )RL(\hat{y},y)$$

where $\hat{y},y$ correspond to the actual and expected outputs respectively, and *α* is greater than zero and less or equal one. determines the contribution of the classification loss to the final loss function. As a result, the model is forced to focus more on finding class instances rather than generating accurate bounding boxes.

## Materials and Evaluation

### Dataset

To acquire necessary data for our experiments, two members of our team visited various sites near Xilinhot, Inner Mongolia, China. They spent 7 days in early August 2018 collecting RGB images of adult grasshoppers using Huawei P20 Pro and Nubia Z11 mini smartphones. Since grasshoppers are very lively insects, some of them were frozen before a photo was taken, although a significant portion of the data set is collected from the wild. The image acquisition process was designed to mirror the expected usage of the mobile application:

1. The majority of images were captured less than 50 centimetres from grasshoppers.
2. Researchers took special care to capture grasshoppers at different angles, poses and living environments of the insects (in grass/on soil).
3. Images were taken in various lighting conditions (shades, different parts of the day).
4. Various foreign objects are present in photo scenes: fingers, boots, gloves etc.

All images were collected between the 11 and 17 August 2018 between the hours of 08:00 to 18:00 every day. Table 3 shows the breakdown of the days and the number of collected images.

**Table 3:** Captured images per day.

Date	Number of images
11/8/2018	152
12/8/2018	382
13/8/2018	910
14/8/2018	879
15/8/2018	536
16/8/2018	517
17/8/2018	202

Roughly 35% of the dataset was collected by shooting frozen grasshopers at CAAS research post near Xilinhot. Grasshoppers are vigilant and lively insects which makes them expert at avoiding focused shots, hence the need for freezing procedure. The frozen grasshoppers are easier to manipulate and facilitate staged shots including multiple insects, obstructions, debris, and shadows.

**Figure 8:**
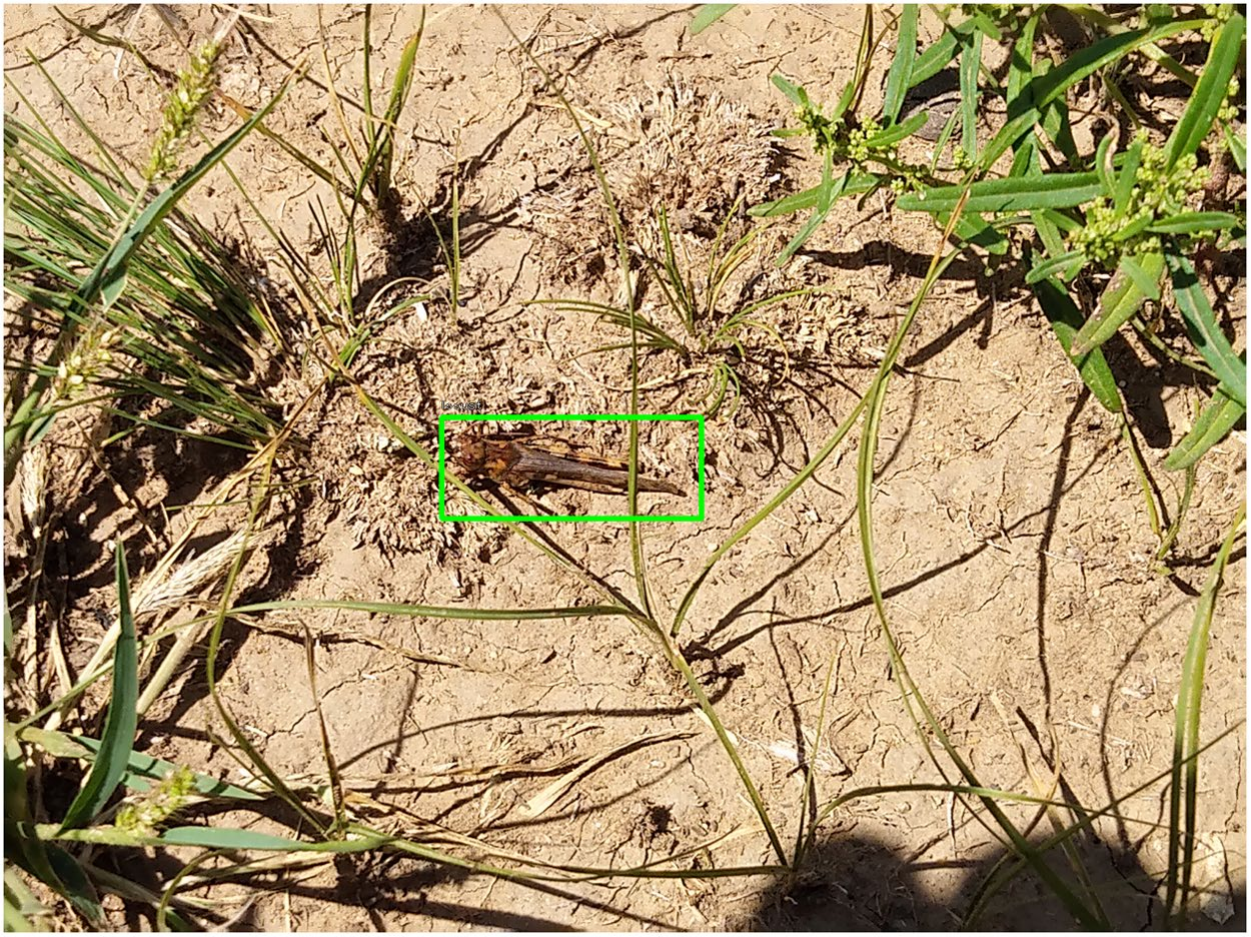
Example image of an adult grasshopper with bounding box annotation from GHCID dataset.

Subsequently, all images were annotated by our grasshopper expert in the UK and were annotated with rectangular bounding boxes in PASCAL VOC format using Labeling tool^32^. To assure high-quality ground truth dataset, the manual approach was adopted where individual images were scrutinised for grasshoppers with approximately 160 hours of total time spent on entire data labelling. All images have been inspected at least twice. The boundary boxes were readjusted where necessary to exclude unwanted foreign objects from the environment and to have minimum box size which includes all the desired details of the insects (the whole body, abdomen, legs, head, antenna, wings). Low-quality images (out of focus, captured more than 50cm from the object, objects obscuring almost whole grasshopper body) were filtered out. Overall, the GHCID dataset consists of 3578 images of 2976 × 3968 resolution with 4406 instances of labelled adult grasshoppers. Figure 8 shows an example grasshopper from GHCID dataset with accompanying annotation. The dataset can be downloaded from the following link: https://lcas.lincoln.ac.uk/owncloud/index.php/s/gGiGUxhYTmPV1vKGHCID.

### Evaluation Metrics

Following other detection works, we use mAP to validate our results^17^. The mAP measures the average of the maximum precisions at different recall values and evaluates the quality of both classification and localization tasks. *Precision* measures the percentage of correct positive predictions. It is the ratio of correctly predicted positive values to the total predicted positive values. High precision indicates a low false positive rate. *Recall*, which is also known as *sensitivity*, measures how good the model is in finding all positive predictions. It is the ratio of correctly predicted positive values to the actual positive values. Both metrics are defined as

2 $$Precision=\frac{TP}{TP+FP},$$

3 $$Recall=\frac{TP}{TP+FN}$$

where TP, FP, FN correspond to true positives (grasshopper detection that are actually grasshoppers), false positives (grasshopper detection that are not grasshoppers) and false negatives (grasshoppers that were not detected) respectively. Apart from precision and recall values, the IoU is required to calculate the mAP. The IoU measures how well the detected region matches the ground truth region (the correctness of the produced bounding box) and is given by

4 $$IoU=\frac{area\,of\,overlap}{area\,of\,union}$$

The average precision (AP) is calculated as the area under the precision-recall graph at 11 recall points [0, 0.1, …, 1] for a specific IoU threshold. The mAP is calculated as the mean AP for all classes (in this case only one class: grasshopper).
