# Author Correction: Mobile Real-Time Grasshopper Detection and Data Aggregation Framework

**DOI:** 10.1038/s41598-020-60909-3

**Published:** 2020-03-10

**Authors:** Piotr Chudzik, Arthur Mitchell, Mohammad Alkaseem, Yingie Wu, Shibo Fang, Taghread Hudaib, Simon Pearson, Bashir Al-Diri

**Affiliations:** 1 The University of Lincoln, School of Computer Science, Lincoln, LN6 7TS UK; 2 The University of Lincoln, Lincoln Institute for Agri-Food Technology, Lincoln, LN6 7TS UK; 3 The University of Lincoln, School of Pharmacy, Lincoln, LN6 7TS UK; 4 State Key Laboratory of Severe Weather, Chinese Academy of Meteorological Sciences, Beijing, 100081 China

Correction to: *Scientific Reports* 10.1038/s41598-020-57674-8, published online 24 January 2020

The original version of this Article contained a typographical error in the Abstract.

“MAESTRO can gather data using cloud storge for further research and in-depth analysis.”

now reads:

“MAESTRO can gather data using cloud storage for further research and in-depth analysis.”

Additionally, the following statement was previously omitted from the Results and Discussion section:

“The mobile App can be downloaded from the following link: https://lcas.lincoln.ac.uk/owncloud/index.php/s/9L0zwZgjsTJMCcn.”

Finally, in the Materials and Evaluation section:

“The dataset can be downloaded from the following link: https://lcas.lincoln.ac.uk/owncloud/index.php/s/gGiGUxhYTmPV1vKGHCID.”

now reads:

“The dataset can be downloaded from the following link: https://lcas.lincoln.ac.uk/owncloud/index.php/s/gGiGUxhYTmPV1vK.”

These errors have now been corrected in the PDF and HTML versions of the Article.

